# Molecular-scale visualization and surface charge density measurement of Z-DNA in aqueous solution

**DOI:** 10.1038/s41598-019-42394-5

**Published:** 2019-05-02

**Authors:** Hiroaki Kominami, Kei Kobayashi, Hirofumi Yamada

**Affiliations:** Department of Electronic Science and Engineering, Kyoto University, Kyoto University Katsura, Nishikyo, Kyoto 615-8510 Japan

**Keywords:** Nanoscale biophysics, Biomaterials

## Abstract

The DNA in the left-handed conformation (Z-conformation) was first discovered by A. Rich, who revealed the crystalline structure of a DNA oligomer d(GC)_3_ by X-ray diffraction method. Later it was also found that DNA molecules change their conformations from typical right-handed form (B-DNA) to the left-handed form (Z-DNA) under specific conditions (B–Z transition). Furthermore, the detailed structures of the interface between B- and Z-DNAs, B-Z junction, was also determined with an atomic resolution. Recently it was found that some proteins have the Z-DNA binding domains, but the biological functions of Z-DNA are not well understood yet. Therefore the investigation of Z-DNA under physiological conditions is highly essential. In this study, we demonstrated the high-resolution real-space imaging of DNA molecules having the Z- and B-form conformations by frequency-modulation atomic force microscopy (FM-AFM), that has made a great progress in recent years, in an aqueous solution. The major and minor grooves of both DNA conformations were clearly visualized. Furthermore, the surface charge density was measured by three-dimensional (3D) force mapping method. We found that Z-form region was less negatively charged than the B-form region.

## Introduction

DNA molecules contain four bases; i.e., adenine (A), thymine (T), guanine (G) and cytosine (C). The complementary DNA molecules form a double-stranded (ds) structure as proposed by Watson and Crick in 1953^1^. Typical dsDNA has a right-handed helical structure referred to as the B-form (B-DNA). While the B-form conformation is the most stable conformation under physiological conditions, some DNA molecules change their conformation under specific conditions^2^. For example, guanine(G)-rich sequences promote the conformation changes of the DNA molecules into various structures such as the Z-form DNA (Z-DNA)^3,4^, triplex DNA^5,6^ and G-quadruplex^7^. It has also been reported that the B-form DNA changes into the Z-form DNA under high ionic concentration conditions^8^ or with the modification of cytosine^9^. Because of this, there is a growing interest in the Z-form DNA. In contrast to the right-handed structure of the B-DNA, the Z-DNA has a left-handed structure^10^. The structures and biological functions of the Z-DNA have been studied for several decades^3,4,11^ since the discovery of the Z-DNA by Rich in 1979^4^. Recently the fine structures of the B–Z junction, a junction of the B-form and Z-form DNAs coexisting in a single dsDNA, was revealed by atomic-resolution X-ray crystallography^12^. It was also revealed that some proteins, such as the double-stranded RNA adenosine deaminase (ADAR1)^13^ and protein kinase^14^, have Z-DNA binding domains. Many researchers have investigated the structure and biological function of the Z-DNA, however, the detailed structures and basic properties in physiological solutions have not been investigated to the best of our knowledge. Since the transition of the B-form into the Z-form (B–Z transition) and the stability of the Z-form conformation under high ionic concentration conditions are related to the difference in the surface charge density of B-form and Z-form dsDNAs^8,15,16^, the surface charge measurement of the B- and Z-form DNAs in physiological solutions is highly demanded.

Atomic force microscopy (AFM)^17–19^ has been widely used to visualize biomolecules in liquids^20,21^. In particular, frequency modulation AFM (FM-AFM)^22^ allows us to obtain high-resolution images of biomolecules such as the double-stranded structures of plasmid DNA^23^ and immunoglobulin G^24^. Although B-DNA molecules and other non-B-form DNA molecules have been visualized by AFM in liquids^23,25^, there have been only a few reports of the AFM study of Z-form DNA molecules in liquids. While there have been several reports on the surface charge density measurements of the B-DNA molecules in aqueous solutions using AFM^26–28^, there has been no experimental report on the surface charge density measurements of the Z-DNA molecules. Here, we visualized the double-stranded structure of Z-form DNA in isolated Z-DNA and DNA having the B–Z junction (B–Z–B DNA), and measured the surface charge density of the B–Z–B DNA in an aqueous solution by FM-AFM.

## Results

### High-resolution imaging of isolated Z-DNA

**Figure 1:**
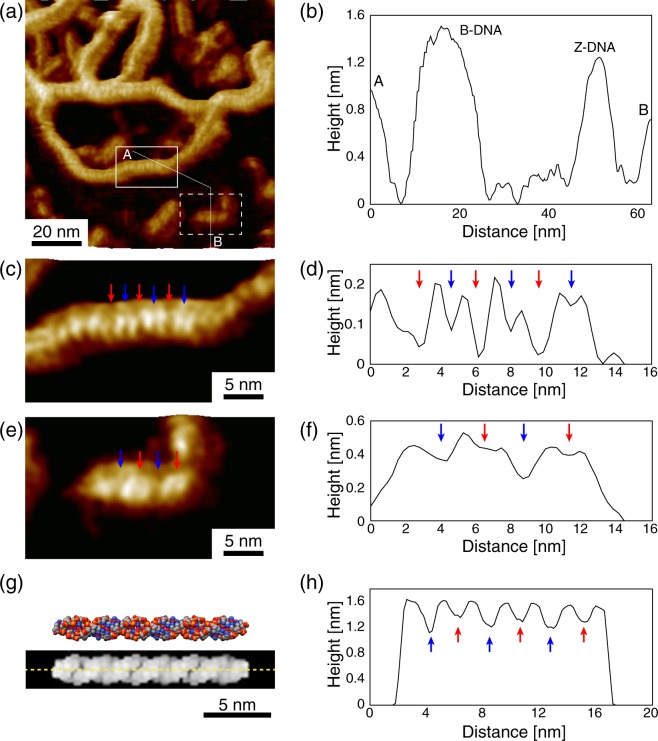
FM-AFM images of B-DNA (plasmid DNA) and Z-DNA. (**a**) Typical FM-AFM image of B-DNA (plasmid DNA) and Z-DNA in 50 mM NiCl_2_ solution. (**b**) Averaged cross-sectional profile along the A–B polyline in (**a**). (**c**) Magnified image of the area indicated by the white solid rectangle in (**a**). (**d**) Averaged cross-sectional profile along the helix axis of the DNA measured in (**a**). (**e**) Magnified image of the area indicated by the white dotted rectangle in (**a**). (**f**) Averaged cross-sectional profile along the helix axis of the DNA measured in (**a**). (**g**) Z-DNA model and simulated AFM image of the Z-DNA using a tip model with a radius of 0.3 nm. (**h**) Averaged cross-sectional profile along the helix axis of the simulated AFM image in (**g**).

Figure 1a shows a topographic image of a plasmid DNA and Z-DNA molecules consisting of d(GC)_36_ and dA_32_ in 50 mM NiCl_2_. In the experiments, the B- and Z-DNAs were adsorbed onto a substrate at the same time to compare the structure of the Z-DNA to that of the B-DNA. We used the high concentration NiCl_2_ solution as the rinsing and imaging solutions such that the DNA oligomers changed their conformations from the B-form to Z-form. A cross-sectional profile along the A–B polyline in Fig. 1a is shown in Fig. 1b. The measured height of the plasmid DNA (B-DNA) was 0.25 nm higher than that of the Z-DNA, which well agrees with the difference in their diameters; the diameter of the B-DNA was 2.0 nm and that of the Z-DNA was 1.8 nm^29^. The measured heights of the molecules were lower than their diameters probably because the tip did not reach the substrate because of the adsorbates as well as the structured water molecules on the substrate. We found periodic features with a different periodicity on the B- and Z-DNA molecules. Figure 1c,d show the enlarged images of the area indicated by the white solid rectangle in Fig. 1a and a cross-sectional profile along the helix axis of the plasmid DNA in Fig. 1c. The helix pitch of the B-DNA was 3.6 nm, which is consistent with a previous study^23^. There are two kinds of grooves; i.e., wide and narrow grooves in one helix pitch. The wide grooves, referred to as the major grooves, are indicated by the red arrows, and the narrow grooves, the minor grooves, are indicated by the blue arrows.

Figure 1e,f show the enlarged images of the area indicated by the white dotted rectangle in Fig. 1a and a cross-sectional profile along the helix axis of the Z-DNA in Fig. 1f. The left-handed structure was clearly resolved. The helix pitch of the Z-DNA was about 4.5 nm, which was consistent with the value measured by X-ray crystallography^27^. We also found shallow deep and shallow grooves in the Z-DNA as shown in Fig. 1f. In order to interpret the result, we simulated the AFM image of the Z-DNA using a tip model with a radius of 0.3 nm. A molecular model of the Z-DNA is shown in Fig. 1g. The simulated AFM image for this molecular model is presented below. It has been revealed by the X-ray diffraction that there is a deep groove in the Z-DNA which is an analogue of the minor groove of the B-DNA^3^, while the groove analogous of the major groove of the B-DNA is very shallow. Hereafter they are referred to as the minor and major grooves of the Z-DNA, respectively. Figure 1h shows a cross-sectional profile along the helix axis, indicated by the dotted line, of the simulated AFM image, which shows the deep (minor) and shallow (major) grooves within a helix pitch, as indicated by the blue and red arrows, respectively.

**Figure 2:**
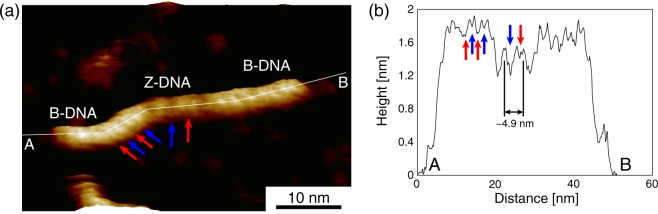
FM-AFM image of the DNA having B–Z junctions. (**a**) High-resolution FM-AFM image of the DNA having B–Z junctions in the 50 mM NiCl_2_ solution. (**b**) Cross-sectional profile along the A–B polyline in (**a**). The red and blue arrows indicate the major and minor grooves, respectively.

A high-resolution FM-AFM image of a DNA having the B–Z junctions with methylation (B–Z–B DNA) is shown in Fig. 2a. The helical structures of the B-form dsDNA and Z-form dsDNA were clearly visualized in both the Z-form part in the center and the B-form parts at both ends, respectively. A cross-sectional profile along the A–B polyline is shown in Fig. 2b. The red and blue arrows in Fig. 2a,b indicate the major and minor grooves of the B-DNA and Z-DNA, respectively. The helical pitch of the Z-DNA is about 4.9 nm that well corresponds to the isolated Z-DNA (Fig. 1e,f). The width of the deep groove is 3.2 nm and that of the shallow groove is 1.7 nm. In addition, the difference between the heights of the B-DNA and Z-DNA is about 0.3 nm, which is also consistent with the results shown in Fig. 1b.

### Surface charge density measurements of Z-DNA

**Figure 3:**
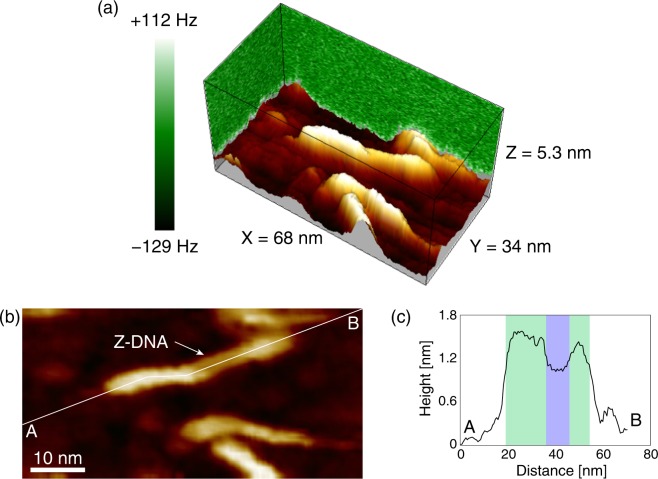
3D frequency shift map measured for DNA having B–Z junctions. (**a**) Representation of 3D frequency shift map of the DNA having B–Z junctions in a 50 mM NiCl_2_ solution. (**b**) Constant ∆*f* image (corresponding to topographic image) at +110 Hz reconstructed from the 3D frequency shift map. (**c**) Averaged cross-sectional profile along the A–B polyline in (**b**). The green and blue shaded areas correspond to the B- and Z-DNA regions, respectively.

We performed the 3D frequency shift mapping using FM-AFM on the B–Z–B DNA molecule. Since the B–Z–B DNA molecules with methylation often caused unexpected aggregations, the unmethylated B–Z–B DNA molecules were used in this experiment to improve the reproducibility. The volume in which the frequency shift mapping was performed is shown in Fig. 3a (X = 68 nm, 128 pixels, Y = 34 nm, 64 pixels, Z = 5.3 nm, 240 pixels). A movie file of the collected 3D frequency shift map is available as the Supplementary Movie. Figure 3b shows the constant ∆*f* image (∆*f* = +110 Hz) reconstructed from the 3D frequency shift map, which corresponds to the topographic image. A cross-sectional profile along the A–B polyline in Fig. 3b is shown in Fig. 3c. The profile shows that the height in the middle region (green shaded region) of the DNA was lower than that of the outer regions of the DNA (blue shaded region), suggesting that the DNA conformations in the middle part was Z-DNA, while the outer regions were B-DNA. In spite of cytosine consisting of the Z-DNA region not being methylated, the B–Z transition was confirmed because of high ion concentration condition.

## Discussion

We obtained the 3D map of the frequency shift in the volume including the interface of the B–Z–B DNA and the solution. Although the surface charges are screened by the surrounding counter ions in aqueous solutions, which form an electric double layer (EDL), the surface charge density can be measured by analyzing the EDL force between the tip and sample. We first converted the recorded frequency shift curves to the force curves by using the Sader method^30^ to obtain the 3D force map, which is a collection of the site-specific force versus distance curves between the tip and sample. We used a simple equation of the EDL force between two spheres assuming the tip and DNA as spheres to estimate the surface charge densities of the DNA under the tip, and calculated the linear charge density of the DNA. The surface charge density of the silicon oxide tip (*σ*_t_) in the aqueous solution used in this experiment was assumed to be −1.8 mC/m^2^ ^31^. On the other hand, the surface charge density of the B- and Z-DNAs (*σ*_s_) should be on the order of −100 mC/m^2^ when the phosphate groups are fully dissociated^26^. Therefore we used the equation of the EDL force between the tip and sample under the condition that the surface charge density of the DNA is much higher than that of the tip (*σ*_s_ ≫ *σ*_t_),

1 $${F}_{{\rm{E}}{\rm{D}}{\rm{L}}{\rm{\_}}{\rm{s}}{\rm{s}}}(z)=\frac{2\pi }{\varepsilon {\varepsilon }_{0}}\frac{{R}_{{\rm{s}}}{R}_{{\rm{t}}}}{{R}_{{\rm{s}}}+{R}_{{\rm{t}}}}(\frac{{\sigma }_{{\rm{s}}{\rm{p}}}^{2}{e}^{-\frac{2z}{{\lambda }_{{\rm{D}}}}}}{1-{e}^{-\frac{2z}{{\lambda }_{{\rm{D}}}}}})$$

where *R*_s_ and *R*_t_ are the radii of the DNA molecule and the tip, respectively, *λ*_D_ is the Debye length, *ε* is the relative dielectric constant, *ε*_0_ is the dielectric constant of a vacuum, and *z* is the distance between the two spheres.

**Figure 4:**
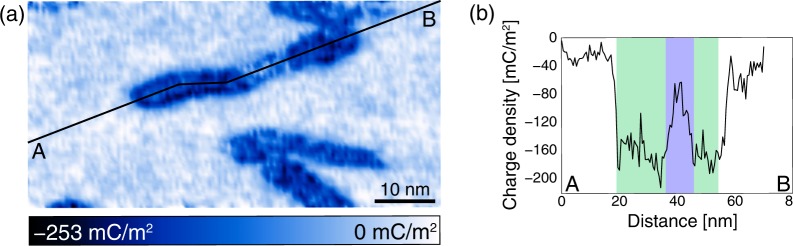
Surface charge density measurement of the B-DNA and Z-DNA. (**a**) Surface charge density map obtained by fitting Eqs (1) and (2) to the experimental force curves. (**b**) Averaged surface potential profile along the A–B polyline in (**a**). The green and blue shaded area corresponds to the B- and Z-DNA regions, respectively.

We set *R*_s_ as 1 nm for the data on the B-DNA and 0.9 nm for the data on the Z-DNA. The tip radius (*R*_t_) was assumed to be 7 nm (manufacturer value). We neglected the contribution of the EDL force between the tip and the substrate to the force curves recorded on the DNA because the tip–substrate distance (DNA diameter) was higher than the Debye length (0.79 nm) in the 50 mM NiCl_2_ solution. Therefore, we only considered the EDL force between the tip and the DNA. For the force curves recorded on the substrate, we used the equation of the EDL force between the spherical tip and the substrate plane,

2 $${F}_{{\rm{E}}{\rm{D}}{\rm{L}}{\rm{\_}}{\rm{s}}{\rm{p}}}(z)=\frac{2\pi {R}_{{\rm{t}}}}{\varepsilon {\varepsilon }_{0}}(\frac{{\sigma }_{{\rm{s}}{\rm{p}}}^{2}{e}^{-\frac{2z}{{\lambda }_{{\rm{D}}}}}}{1-{e}^{-\frac{2z}{{\lambda }_{{\rm{D}}}}}}),$$

which is the reduced form of Eq. (1) under the condition *R*_s_ ≫ *R*_t_. We obtained the surface charge density map by fitting Eqs (1) and (2) to force curves on the DNA molecule and the surface, respectively, as shown in Fig. 4a. To exclude the contribution of the van der Waals force, the non-shaded region was used for the fitting (see Supplementary Fig. 2 for the original force curves). Figure 4b shows a surface potential profile along the A–B polyline in Fig. 4a. It was clearly found that the surface charge density of the B-DNA was greater than that of the Z-DNA. We averaged force curves on the B-DNA and Z-DNA, and determined the surface charge density of the B-DNA and Z-DNA as −163 mC/m^2^ and −116 mC/m^2^, respectively. Note that the surface charge density of the B-DNA was in quite good agreement with a theoretical value (–150 mC/m^2^) considering that the phosphate groups are fully dissociated^26^.

We consider that the difference in the surface charge density depending on the conformation predominantly originates from the differences in the helical pitch. Since the Z-DNA molecules have slightly stretched structure compared with the B-DNA, the distance between base pairs for Z-DNA is higher than that for B-DNA. In the previous studies, the linear charge densities of B-DNA and Z-DNA were estimated by assuming the full dissociation of the phosphate groups^15,16^; Two elementary charges per base pair length (0.34 nm and 0.37 nm) gives the linear charge densities of B-DNA and Z-DNA as −942 pC/m and −866 pC/m, respectively^15^. In this case, the linear charge density of Z-DNA is lower than that of B-DNA by about 8%. Since the linear charge density of a cylinder having a radius of *R*_s_ and a surface charge density of *σ*_s_ is calculated as 2*πR*_s_*σ*_s_, the linear charge densities of the B- and Z-DNAs in our measurement are calculated as −1024 pC/m and −656 pC/m, respectively. Our measurement showed that the linear charge density of Z-DNA is lower than that of B-DNA by about 36%. One of the possible reasons for the discrepancy is the difference in the location of the negatively charged oxygen. The distance of the charged oxygen from the center is reported as 0.95 nm and 0.715 nm for B- and Z-DNAs, respectively^15^, and the EDL force measured at the molecular surface is smaller when the charge is located deeper in the molecule.

We demonstrated the molecular-scale visualization and the surface charge density measurement of the Z-DNA in an aqueous solution by using FM-AFM. The isolated short Z-DNA as well as the plasmid DNA were clearly visualized. The deep and shallow grooves of the Z-DNA were differentiated. We also visualized a DNA having the B–Z junctions with methylation. We again observed the characteristic left-handed helical structures of the Z-form DNA. We then performed a 3D force mapping on the DNA oligomer containing the B-form region and Z-form region. By detecting the EDL forces, the linear charge density of the Z-DNA was measured as −656 pC/m, which is 64% of the B-DNA value.

We believe that these results lead to understanding of the function of the DNA molecules, not only the Z-DNA but also the B-DNA, since the conformation and the surface charge density play an important role in the DNA–protein recognition. The conformational change of the DNA molecules induced by guanine-rich sequence is related to the biological functions of the DNA such as gene expression and regulation. Therefore, it is important to visualize the structure of non B-DNA conformations for understanding the functions of the DNA in biological systems. The results presented are the first demonstration of the capability of AFM to measure the surface charge density distribution in a single biomolecule. Therefore, AFM can be used for nanometer-scale investigations of structures and basic properties of biomolecules and their complex, such as the DNA–protein complex, in physiological solutions.

## Methods

### Isolated Z-DNA and plasmid DNA

Single-stranded DNA oligomers consisting of dA_32_ and d(GC)_36_, were commercially synthesized by BEX. The DNA oligomers were dissolved in a TNE buffer solution (10 mM Tris-HCl, 1 mM EDTA, 150 mM NaCl, pH 8.0) at a concentration of 9 µM. The oligomer solution was heated to 77 °C, then cooled to 20 °C over 1 hour. After annealing, the DNA solution was diluted to a concentration of 90 nM with a TE buffer solution. The plasmid DNA (pUC18, 2686 base pairs) molecules were purchased from Takara Bio. The plasmid DNA molecules were dissolved in the TE buffer to a concentration of 5 ng/µl. Five µl droplets of the Z-DNA solution, plasmid DNA solution, and a solution containing 50 mM nickel(II) chloride hexahydrate (NiCl_2_·6H_2_O, 99.9998% purity, Alfa Aesar) were deposited onto a fleshly cleaved muscovite mica substrate (Furuuchi Chemical). After waiting for five minutes, the substrate was rinsed by the 50 mM NiCl_2_ solution, and imaged in the same solution without drying the sample. The UCSF Chimera package (Resource for Biocomputing, Visualization and Informatics, University of California, San Francisco)^32^ was used to generate a graphical representation of the Z-DNA (Fig. 1(g)).

### DNA having B-Z junctions with methylation

Complementary single-stranded DNA (ssDNA) oligomers each having d(G^5me^C)_12_ at the center and a random sequence at both ends were commercially synthesized by Eurofins Genomics (see Supplementary Information). Cytosine bases in poly d(G^5me^C)_12_ sequences at the center were methylated because methylation of the cytosine makes the Z-form conformation stable under physiological conditions^33^. The DNA oligomers were dissolved in the TE buffer solution to a final concentration of 2 µM. The solution containing the DNA oligomers was diluted with the TNE buffer solution to a concentration of 20 nM. The DNA solution was heated to 80 °C and slowly cooled to 40 °C over 12 hours. After annealing, the DNA solution was dropped onto a fleshly cleaved mica substrate and imaged in the same manner as already mentioned.

### DNA having B-Z junctions without methylation

The complementary ssDNA consisting of d(GC)_12_ and the random sequence were commercially synthesized by Eurofins Genomics (see Supplementary Information). The DNA oligomers were dissolved in the TE buffer solution to a concentration of 10 µM. The DNA solution was diluted with the TNE buffer solution to a concentration of 40 nM. The solution containing the DNA oligomers was heated to 85 °C and slowly cooled to 20 °C at the rate of −1 °C/min. After annealing, the DNA solution was dropped onto a fleshly cleaved mica substrate and imaged in the same manner as already mentioned.

### FM-AFM imaging

We used lab-modified FM-AFM instruments based on a Shimadzu SPM-9600 with a home-build controller programmed in LabVIEW (National Instruments). A silicon cantilever (OMCL-AC240TN, Olympus), whose nominal second spring constant and resonance frequency in the imaging solution were 91 N/m and 150 kHz, respectively, was used. The cantilever was oscillated at its second resonance frequency and the frequency shift was detected by a digital phase-locked loop (HF2LI, Zurich Instruments). The typical oscillation amplitude was 0.5 nm peak-to-zero. WSxM (Nanotech Electronica)^34^ was used to analyze the obtained data.

### Simulation of topographic AFM images

GeomAFM Simulator software (version 1.1) in the SPM SimSoftware Suite^35^ was used to simulate the AFM image of the Z-DNA. The AFM image was simulated by calculating the tip trajectory when the tip touched and followed the outermost atoms. The tip was modeled as a sphere with a radius of 0.3 nm and a cone with a half cone angle of 10°. The molecular structure was obtained from the Protein Data Bank (PDB ID code: 2DCG^4^).

## Supplementary information

Supplementary Info

BZ-DNA_charge_withtopo
