# Supplementary material for: Molecular-scale visualization and surface charge density measurement of Z-DNA in aqueous solution
Source: Sci Rep. 2019 May 2;9:6851. doi: 10.1038/s41598-019-42394-5 (PMC6497900; doi:10.1038/s41598-019-42394-5)
Supplement: Supplementary file 1 — Supplementary Info [file 41598_2019_42394_MOESM1_ESM.pdf]

Supplementary information

Molecular-scale visualization and surface charge density measurement of Z-DNA in aqueous solution

Hiroaki Kominami, Kei Kobayashi, Hirofumi Yamada

Department of Electronic Science and Engineering, Kyoto University, Katsura, Nishikyo, Kyoto, 615-8510, Japan

Correspondence and requests for materials should be addressed to H.Y. (email: h-yamada@kuee.kyoto-u.ac.jp).

(a)

GCGCGCGCGCGCGCGCGCGCGCGCGCGCGCGCGCGCAAAAAAAAAAAAAAAAAAAAAAAAAA

(b)

TACCGAGCTCGAATTCGTAATCATGGTCATAGCTGTTTCCTGTGTGCGCGCGCGCGCGCGCGCGCGCTGTTATCCGCTCACAATTCCACACAACATACGAGCCGGAAGCATA
TATGCTTCCGGCTCGTATGTTGTGTGGAATTGTGAGCGGATAACAGCGCGCGCGCGCGCGCGCGCGCACACAGGAAACAGCTATGACCATGATTACGAATTCGAGCTCGGTA

(c)

TACCGAGCTCGAATTCGTAATCATGGTCATGCGCGCGCGCGCGCGCGCGCGCATTGTTATCCGCTCACAATTCCACACAACATACGAGCCGGAAGCAT
ATGCTTCCGGCTCGTATGTTGTGTGGAATTGTGAGCGGATAACAATGCGCGCGCGCGCGCGCGCGCGCATGACCATGATTACGAATTCGAGCTCGGTA

Supplementary Figure 1 | Sequences of DNA oligomers. a, Isolated Z-DNA. **b,** DNA having B–Z junctions with methylation. **c,** DNA having B–Z junctions without methylation

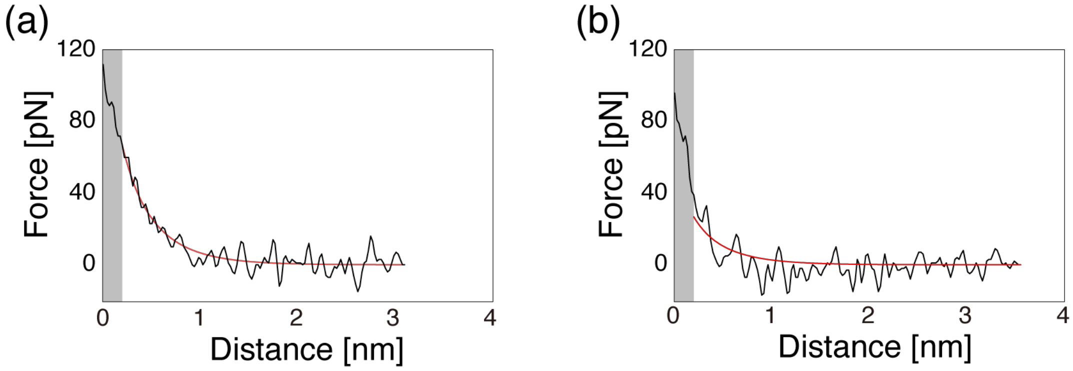

Supplementary Figure 2 | Averaged force curves. a, Averaged force curve measured on B-DNA. **b,** Averaged force curve measured on Z-DNA. Non-shaded regions were used for fitting.

Legend for Supplementary Movie

Movie 1. 3D frequency shift map obtained by FM-AFM. 3D frequency shift map which was obtained on DNA oligomers for surface charge density measurement of B-DNA and Z-DNA
